# Sialylation of *Helicobacter bizzozeronii* lipopolysaccharides modulates Toll-like receptor (TLR) 2 mediated response

**DOI:** 10.1186/s13567-014-0133-4

**Published:** 2015-01-21

**Authors:** Pradeep Kumar Kondadi, Joana Revez, Marja-Liisa Hänninen, Mirko Rossi

**Affiliations:** Department of Food Hygiene and Environmental Health, Faculty of Veterinary Medicine, University of Helsinki, P.O. Box 66, Agnes Sjöbergin katu 2, FI-00014 Helsinki, Finland

## Abstract

Sialic acid in lipopolysaccharides (LPS) of mucosal pathogens is known to be an important virulence factor. Few strains of *Helicobacter pylori* express sialyl-Lewis-X and we have reported that human and canine *Helicobacter bizzozeronii* strains express sialyl-lactoseamine in their LPS. However, the role of sialyation of *Helicobacter* LPS in the interaction with the host cells is still unknown. In this study *H. bizzozeronii* LPS is shown to activate the TLR2 in a dose and strain dependent manner in the in vitro HEK-293 cells model expressing TLR2, but not the cells expressing TLR4. These results indicate that TLR2 is the specific receptor for *H. bizzozzeronii* LPS, as previously described for *H. pylori*. To further explore the role of sialylation of *H. bizzozeronii* LPS on TLR2 response, *H. bizzozeronii Δhbs2* mutant strains deficient in sialyltransferase activity were constructed by homologous recombination. LPS from *H. bizzozeronii Δhbs2* strains enhanced the NF-ĸB induction via TLR2 compared to the respective wild types, leading to the conclusion that the sialylation of *H. bizzozeronii* LPS in wild-type strains may modulate host immune response.

**Electronic supplementary material:**

The online version of this article (doi:10.1186/s13567-014-0133-4) contains supplementary material, which is available to authorized users.

## Introduction

In both humans and several animal species helicobacters are recognised as an etiologic agent of chronic gastritis, gastric ulcers and, in some cases, even gastric adenocarcinoma and MALT lymphoma [1,2]. *Helicobacter* spp. are highly diverse, appear to have a host species-specific tropism, the transmission occurs mainly vertically and the colonisation generally persists throughout the lifetime of their hosts [1,2]. Several species have also developed the ability to jump between different hosts [1,3]. Among them, the gastric *Helicobacter* spp., belonging to the group of *H. heilmannii* sensu lato (including *H. bizzozeronii*, *H. felis*, *H. suis* and *H. heilmannii* sensu stricto), have zoonotic potential and have been detected in 0.6-2% of human gastritis [1,3].

After penetration of the mucous layer, gastric epithelial cells provide the first point of contact between helicobacters and their hosts [4]. By recognizing diverse microbial products, Toll-like receptors (TLRs) expressed on the surface of gastric epithelial cells play a key role in mediating cell-signalling which results in the induction of host defence stimulation [5]. Although all described TLRs have the ability to activate the key transcription factors NF-κB and AP-1, there are differences in the ultimate gene-expression profile that results from the activation of a specific TLR [6]. For example, it has been described that TLR2 and TLR4 differentially activate human dendritic cells (DC) resulting in differences in cytokine and chemokine gene transcription, suggesting that TLR2 and TLR4 signalling is not equivalent [7]. TLR4 specifically recognizes Lipopolysaccharide (LPS) from Gram negative bacteria [8]. TLR2 has a broader spectrum of ligands including also forms of LPS that are structurally different from those recognized by TLR4 [6,8]. In particular, it has been described that *H. pylori* LPS specifically stimulate TLR2 and act as antagonist of TLR4 [6,9]. Very little is known about the ability of non-*H. pylori Helicobacter* species LPS in modulating host-response, despite the fact they express different LPS structures [10,11]. Actually, the human pathogen canine-adapted *H. bizzozeronii* expresses phase variable sialyl-lactoseamine [11]: a feature not detected in *H. pylori* which rarely express sialyl-Lewis antigens on their LPS. Therefore, the aim of this paper was to elucidate the interaction between *H. bizzozeronii* LPS and human TLR as first stage in host-bacterial recognition, focusing in particular on understanding the role of sialylated LPS in this interaction.

## Material and methods

### Cell lines

HEK-293 cells from InvivoGen (Toulouse, France) stably transfected with human TLR-2 (HEK-Blue™-hTLR2), and TLR4 (HEK-Blue™-hTLR4) and HEK-Blue™ Null1 cells non-expressing either TLR2 or TLR4 were grown and maintained according to the manufacturer’s guidelines. Briefly, HEK-Blue™- cell lines were cultured in DMEM (Life Technology, Carlsbad, CA, USA) containing 10% Fetal Bovine Serum (FBS) supplemented with 50 U.mL^−1^ of penicillin and 50 μg.mL^−1^ of streptomycin (Life Technology), 100 μg.mL^−1^ of normocin (InvivoGen), and 1X HEK-Blue™ selection (InvivoGen; selection for HEK-Blue™-hTLR2 and HEK-Blue™-hTLR4) or 100 μg.mL^−1^ zeocin (InvivoGen; selection for HEK-Blue™ Null1).

### Bacterial strains and growth conditions

Bacterial strains used in this study are listed in Table 1. *Helicobacter* strains were routinely grown on HP medium (LabM Limited, Lancashire, UK) containing 5% bovine blood and Skirrow selective supplement (Oxoid, Ltd., Cambridge, UK) at 37 °C in an incubator with microaerobic atmosphere (Thermo Forma, Series II Water Jacketed Incubator; Thermo Fisher Scientific, Waltham, MA, USA). For LPS extraction *Helicobacter* strains were cultivated in Brain Heart Infusion (BHI, BD, Becton, Dickinson and Co., NJ, USA) containing 10% of FBS, Skirrow selective supplement (Oxoid) and Vitox supplement (Oxoid) at 37 °C in a jar with microaerobic atmosphere. *Campylobacter jejuni* was cultivated in Nutrient Agar (Oxoid) supplemented with 5% of bovine blood.

**Table 1:** **Bacterial strains used in this study (h: human isolates; c: canine isolates).**

**Bacterial species**	**Strain**	**Reference or source**
*Helicobacter bizzozeronii*	CIII-1^GEN^ (h)	[12,13]
	R53 (h)	[14]
	Storkis CCUG 35545^T^ (c)	[15]
	14 CCUG 35546 (c)	[15]
	12a (c)	[15]
	10 F (c)	[15]
	Yrjälä (c)	[15]
	Emo (c)	[15]
	Heydar (c)	[15]
	Heydar Δ *hsb2*-M2	this study
	Heydar Δ *hsb2*-M4	this study
	CIII-1^GEN^ Δ *hsb2*-M9	this study
	CIII-1^GEN^ Δ *hsb2*-M13	this study
*Helicobacter pylori*	26695	[16]
*Campylobacter jejuni*	81-176	[17]

### *H. bizzozeronii* α2,3-sialyltransferase gene (*hbs2)* mutant strains

In *H. bizzozeronii* strains CIII-1 (human isolate) and Heydar (canine isolate), chromosomal inactivation of α2,3-sialyltransferase gene (*hbs2*) was performed by allelic exchange using the chloramphenicol resistance gene (*cat*), as previously described [18]. The *cat* gene was introduced in the same direction as the target gene using *Xba*I and *Kpn*I restriction sites. The resultant plasmid, pMRS3, was constructed and amplified in *E. coli* TOPO10 and used as a suicide plasmid in *H. bizzozeronii*. Mutants were obtained by electroporation as described for *H. felis* [19]. After electroporation, the bacteria were left to recover on HP agar plates for 48 h under microaerobic conditions as described above. The mutant strains were selected on HP agar plates supplemented with 10 mg.mL^−1^ of chloramphenicol (Sigma-Aldrich, St. Louis, MI, USA). The plates were incubated for up to 10 days, and the site of recombination was verified by PCR and sequencing.

### LPS extraction and LPS SDS-PAGE profile

LPS was extracted from biomass obtained after 48 h of incubation in BHI broth. Crude LPS was extracted by using the hot phenol-water method, and subsequent purification by enzymatic treatments (RNase A, DNase II and proteinase K) as described previously [6]. LPS were treated with Lysozyme to remove traces of peptidoglycan contamination [6]. After the enzymatic treatments, the LPS was precipitated at −20 °C overnight in 10 volumes of pure ethanol in presence of 0.03 M of sodium acetate and re-suspended in water and the concentration was then determined by purpald assay [20]. The LPS obtained was essentially free of proteins and nucleic acids, and it had an electrophoretic profile similar to that previously reported for the low-molecular-mass *H. bizzozeronii* LPS [11]. LPS was treated overnight with 6.7 U.mL^−1^ of neuraminidase from *Clostridium perfringens* (Sigma-Aldrich) at pH 6. LPS neuraminidase treated and untreated samples were loaded on 15% TRIS-Glycine SDS-PAGE (Biorad, Hercules, CA, US), run for 2 h and 50 min at constant 20 mA and then silver stained as previous described [11].

### NF-ĸB stimulation using HEK-Blue™ system and IL-8 determination

HEK-Blue™ cell lines are engineered HEK293 cells stably transfected with a vector expressing secreted embryonic alkaline phosphatase reporter (SEAP) gene under the control of an inducible NF-ĸB promoter. Therefore, stimulation of TLRs will result in an amount of extracellular SEAP in the supernatant that is proportional to the level of NF-ĸB induction. For the determination of NF-ĸB stimulation, 96-well plates were seeded with 5.0 × 10^4^ cells/well of HEK-Blue™-hTLR2 or HEK-Blue™ Null1, or 2.5 × 10^4^ cells/well of HEK-Blue™-hTLR4. After overnight incubation, cells were treated for 24 h with different concentrations of LPS extracted from *H. bizzozzeronii* strains. SEAP was measured at OD_620_ after 1 h of incubation at 37 °C, by the addition of 180 μL of QUANTI-Blue™ (InvivoGen) to 20 μL of the HEK-Blue™ cells supernatants [21]. For some experiments LPS was pre-treated with 40 μg.ml^−1^ polymyxin B sulphate (PB; Sigma-Aldrich) at 37 °C for 45 min. As positive control for HEK-Blue™-hTLR2, HEK-Blue™-hTLR4 and HEK-Blue™ Null1, 100 ng.mL^−1^ of Pam2CSK4 (Invivogen), 5 ng.mL^−1^ of *E. coli* LPS (Sigma-Aldrich) and 50 ng/mL of Polyinosinic-polycytidylic acid (Invivogen) were used, respectively. After 24 h incubation, induced HEK-Blue™-hTLR2 supernatant was collected and IL-8 expression was determined by ELISA according to the manufacturer’s guidelines (DuoSet ELISA development system: R&D systems). All experiments were done in triplicate.

### Statistical analysis

Statistical analysis was performed using GraphPad Prism version 6 for Windows, (San Diego California USA). For groups comparison one-way ANOVA followed by Bonferroni post-test with a cut-off of 0.05 was selected, while for pairwise comparison unpaired two-tailed *t*-test was carried out. For trend analysis one-way ANOVA was followed by the test for linear trend as implemented in the software. Error bars in the graphs in all figures were calculated as Standard Error of the Mean (SEM).

## Results

**Figure 1:**
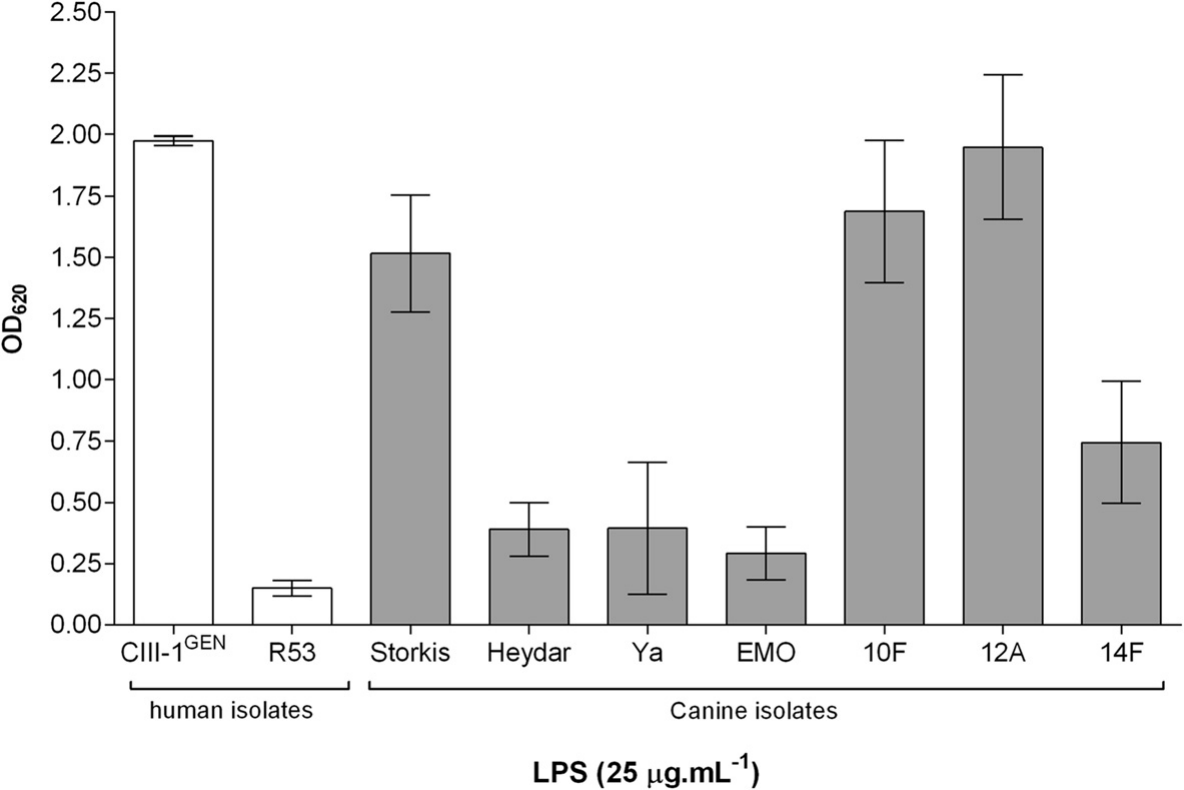
**Differential human TLR2 activation by** ***H. bizzozeronii*** **LPS.** HEK-Blue™-hTLR2 cells were incubated for 24 h with LPS (25 μg.mL^−1^) extracted from different human (white bars) and canine (grey bars) strains, and NF-κB-induced SEAP activity was assessed using QUANTI-Blue™ and by reading the OD at 620 nm. The values were normalized by subtracting the average OD_620_ values of the untreated cells. The OD_620_ value of the positive control Pam2CSK4 was 2.25 ± 0.12. Error bars show ± SEM.

**Figure 2:**
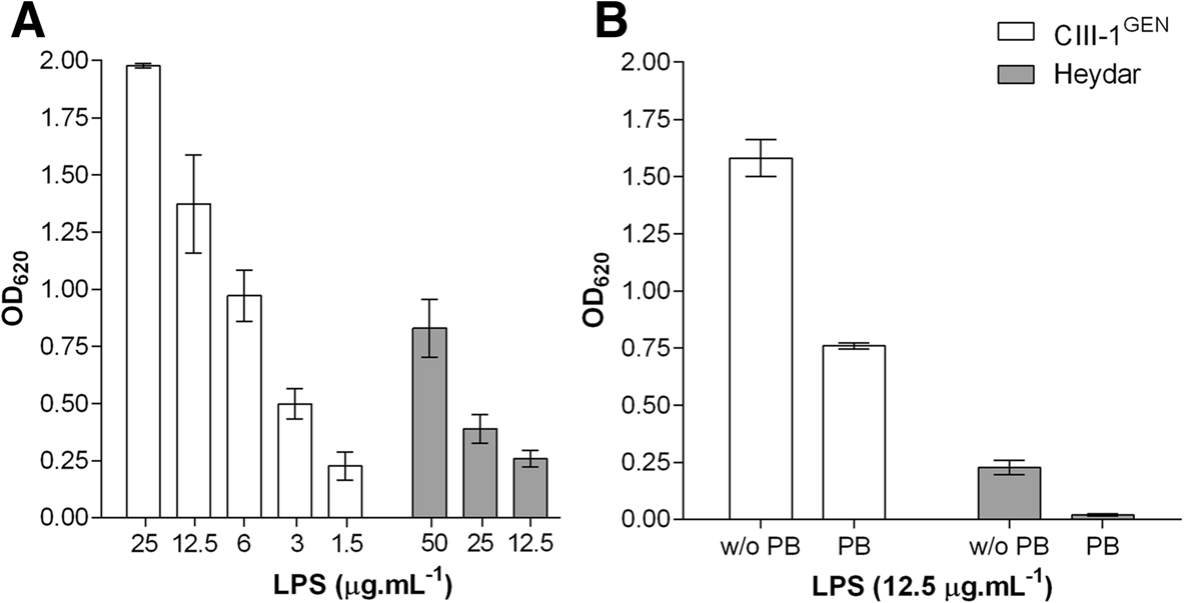
**Human TLR2 response is mediated by** ***H. bizzozzeronii*** **LPS** ***.*** **(A)** *H. bizzozeronii* LPS activates human TLR2 in dose dependent manner. HEK-Blue™-hTLR2 cells were incubated for 24 h with different concentrations of LPS from human isolate CIII-1^GEN^ (white bars) and canine isolate Heydar (grey bars) *H. bizzozzeronii* strains. HEK-Blue™-hTLR2 response decrease significantly (test for linear trend; *p* < 0.05). Error bars show ± SEM. **(B)** Inhibitory effect of Polymyxin B in TLR2 activation by *H. bizzozzeronii* LPS. LPS (12.5 μg.mL^−1^) extracted from the human strain CIII-1^GEN^ (white bars) and the canine Heydar (grey bars) *H. bizzozzeronii* strains, were pre-incubated with PB (40 μg.mL^−1^) for 45 min at 37 °C. HEK-Blue™-hTLR2 cells were incubated with LPS (with and without PB treatment) for 24 h. HEK-Blue™-hTLR2 response was inhibited in both strains after treatment with PB and the response was significantly different (*p* < 0.05) in both the strains. The values were normalized by subtracting the average OD_620_ values of the untreated cells. The OD_620_ value of the positive control Pam2CSK4 was 2.25 ± 0.12. Error bars show ± SEM.

**Figure 3:**
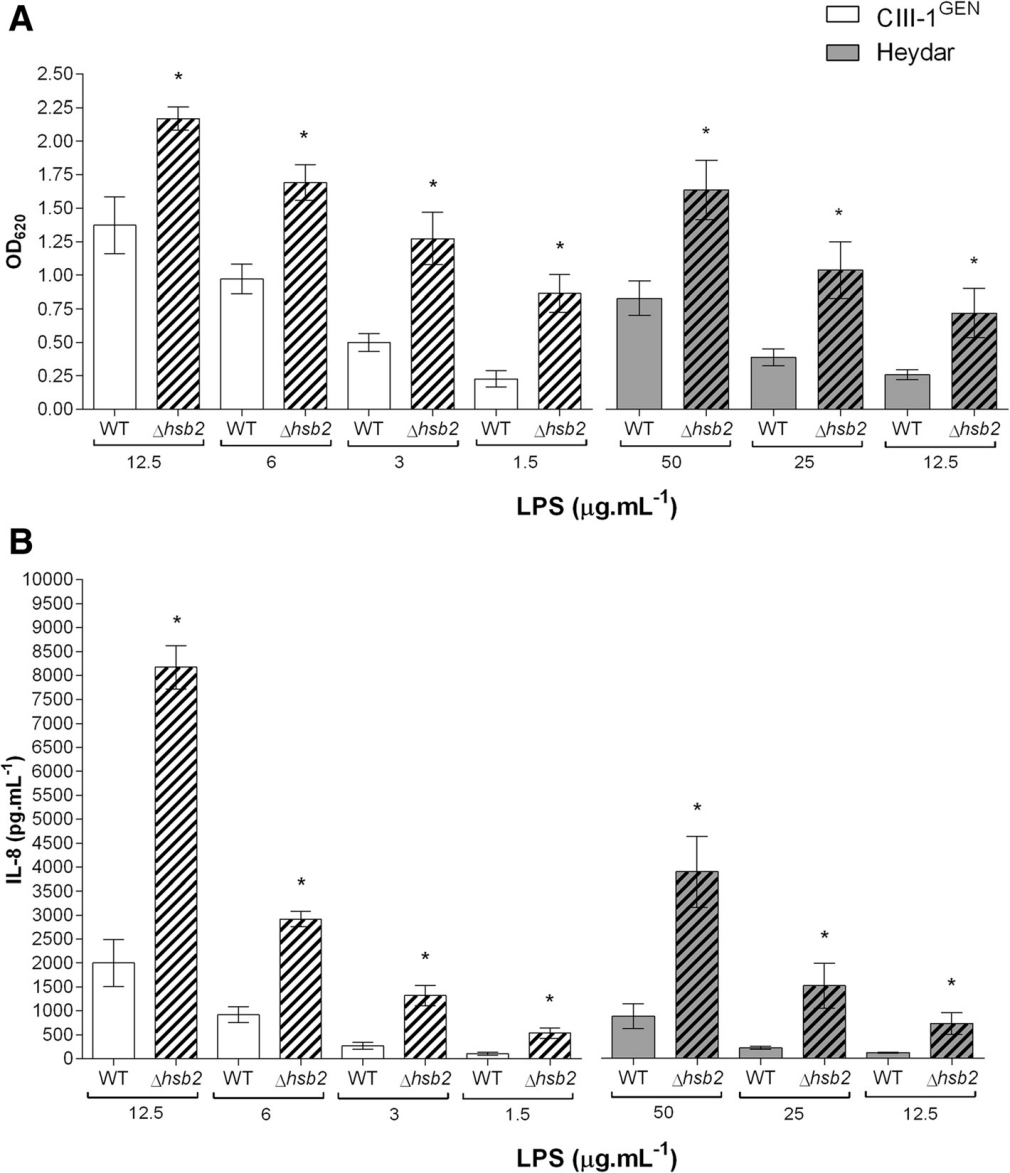
**Desialylated LPS of** ***H. bizzozeronii*** **increases human TLR2 response. (A)** NF-ĸB stimulation and **(B)** IL-8 expression by HEK-Blue™-hTLR2 cells were determined after 24 h of incubation with different concentrations of LPS isolated from CIII-1^GEN^ wild type (white bars), Heydar wild type (grey bars) and respective Δ*hbs2* mutants (striped bars). The TLR2 response between wild type and mutants was significantly different (*p < 0.05), respectively. Error bars show ± SEM.

To identify the TLR responsible for the recognition of *H. bizzozzeronii* LPS, HEK-293 cells stably transfected with human TLR-2 (HEK-Blue™-hTLR2), and TLR4 (HEK-Blue™-hTLR4) and HEK-Blue™ Null1 cells non-expressing either TLR2 or TLR4 were stimulated with 25 μg.mL^−1^ of LPS extracted from several human and canine *H. bizzozzeronii* strains. *H. bizzozzeronii* LPS showed to activate only the HEK-Blue™-hTLR2 in a strain dependent manner (Figure 1). The NF- ĸB induction in HEK-Blue™-hTLR4 or HEK-Blue™ Null1 cells treated with *H. bizzozeronii* LPS was not different from untreated cells (OD_620_, 0.108 ± 0.03). For further analysis, two *H. bizzozeronii* strains inducing high (CIII-1^GEN^) and low (Heydar) NF- ĸB were selected. NF- ĸB induction in HEK-Blue™-hTLR2 cells in response to *H. bizzozeronii* CIII-1^GEN^ and Heydar LPS was found to be dose dependent (one-way ANOVA, test for linear trend, *p* < 0.05; Figure 2A). To demonstrate that the observed TLR2-mediated effects were LPS specific, LPS was pre-incubated with the antibiotic polymyxin B sulphate (PB), an inhibitor of the activating properties of LPS [6]. Therefore, 12.5 μg.mL^−1^ of LPS from *H. bizzozzeronii* CIII-1^GEN^ and Heydar were pre-treated with 40 μg.mL^−1^ of PB at 37 °C for 45 min before infecting the HEK-Blue™-hTLR2. A significant difference in the NF-ĸB –inducing ability of LPS before and after treatment was observed for both stains (unpaired two-tailed *t*-test *p* < 0.05; Figure 2B), indicating that TLR2 activation was mediated by *H. bizzozzeronii* LPS. PB was able to inhibit the TLR2 activation only partially. This is a consequence of low extent of phosphorylation in the lipid A of *Helicobacter* spp. LPS which is required for binding PB, as previously described [6]. In order to study the effect of sialylation of *H. bizzozeronii* LPS on TLR2 response, *H. bizzozeronii Δhbs2* mutant strains deficient in sialyltransferase activity were created. The LPS profile of CIII-1^GEN^ wild type and Heydar wild type showed low-molecular-weight LPS with a clear switch of the band after neuraminidase treatment but no switch was observed in the CIII-1^GEN^ Δ*hbs2* and Heydar *Δhbs2* mutants as we expected (see Additional file 1). HEK-Blue™-hTLR2 were incubated for 24 h with different concentrations of LPS isolated from CIII-1^GEN^ wild type, Heydar wild type and corresponding *Δhbs2* mutant strains (Figure 3). *H. bizzozeronii* CIII-1^GEN^ Δ*hbs2*-M9 and Heydar *Δhbs2*-M4 mutant strains enhanced significantly the induction of NF-ĸB compared to wild type strains (unpaired two-tailed *t*-test *p* < 0.05; Figure 3A). To further explore IL-8–inducing activity of *H. bizzozeronii* LPS, supernatants of HEK-Blue™-hTLR2 were collected after 24 h of incubation with different concentrations of LPS isolated from CIII-1^GEN^ wild type, Heydar wild type and corresponding *Δhbs2* mutant strains and IL-8 expression was determined by DuoSet ELISA Kit. The disruption of *hbs2* in *H. bizzozeronii* significantly enhanced the expression of IL-8 HEK-Blue™-hTLR2 cells (unpaired two-tailed *t*-test *p* < 0.05; Figure 3B). To verify if that increase of IL-8 expression by HEK-Blue™-hTLR2 was a result of the mutation of *hbs2* and not due to a polar effect, and in the absence of a protocol for the complementation, two independent isogenic mutants were selected one from each *H. bizzozeronii* strains: CIII-1^GEN^ Δ*hbs2*-M13 and Heydar *Δhbs2*-M2. After inoculating HEK-Blue™-hTLR2 cells with 12.5 μg.mL^−1^ of LPS we observed that both isogenic *H. bizzozeronii* CIII-1^GEN^ Δ*hbs2*-M13 and Heydar *Δhbs2*-M2 mutants enhanced the expression of IL-8 by 2.4 and 3.0 fold, compared to the respective wild type strain. Although we observed a lower increase of IL-8 expression compared to *H. bizzozeronii* CIII-1^GEN^ Δ*hbs2*-M9 (5.4 fold) and Heydar *Δhbs2*-M4 (4.8 fold) mutants, these results confirmed the role of core or O-chain composition and particularly the presence of sialic acid in the modulation of the host response through TLR2 activation.

## Discussion

There have been conflicting findings in the literature concerning the TLR responsible for the recognition of *Helicobacter* LPS, as some authors have suggested that *Helicobacter* LPS stimulate TLR4, whereas others proposed a role for TLR2 [6,22]. Although we cannot completely exclude possible contaminations, the absence of NF-ĸB induction in either HEK-Blue™-hTLR4 or HEK-Blue™ Null1 cells, the suppression effect of PB on the TLR2 activation and the dose-depend response of HEK-Blue™-hTLR2 suggest that *H. bizzozeronii* LPS function as a classic TLR2 ligand, as described for *H. pylori* [6] and for *H. felis* [22]. We observed a degree of variation in the TLR2 mediated NF-ĸB –inducing ability of LPS prepared from different strains of *H. bizzozeronii*. As previously described, these differences could be the consequence of variations in the degree of acylation and/or phosphorylation of the LPS from different isolates [6]. However, MALDI-TOF analysis indicated that Lipid A structures from both the selected *H. bizzozeronii* strains CIII-1^GEN^ (inducing high concentration of NF- ĸB) and Heydar (inducing low concentration of NF- ĸB) were identical, resembling that of *H. pylori* [23] in which 3-hydroxyoctadecanoic acid, n-octadecanoic acid, and 3-hydroxyhexadecanoic acid are substituting at O- and N positions (see Additional file 2). In addition, phosphoethanolamine was shown to be present in the Lipid A of both *H. bizzozzeronii* strains and no extra phosphates were detected (data not shown). Therefore, in agreement with previous studies [6,22], we hypothesized that also differences in the core and O-chains of the LPS from individual isolates could modulate the IL-8–inducing activity of the lipid A component [22]. In fact, although both *H. bizzozeronii* CIII-1^GEN^ and Heydar strains have a single copy of α2,3-sialyltransferase gene (*hbs2*) and express sialic acid on their LPS, they shown different reactivity with cholera toxin, indicating possible different LPS structures [11]. Studies on *C. jejuni* have shown that sialylation of lipooligosaccharides (LOS) modulates the dendritic cells (DC) response via TLR4 [24]. In particular, the presence of sialic acid on the *C. jejuni* LOS induced a stronger DC activation and subsequent B cell proliferation than did desialylated LOS by increasing TLR4-mediated signalling [24]. These data together with the results from another study [25] indicate that not only the lipid A structure but also the *C. jejuni* LOS carbohydrate moiety modulates TLR4-mediated host-response. Similarly, in the present study we showed that TLR2-mediated NF-ĸB induction and resulting IL-8 expression in HEK293 was modulated by the *H. bizzozeronii* LPS carbohydrate moiety and in particular by the presence of sialic acid. However, differently to what is observed in TLR4, sialylation seems to inhibit TLR2-mediated induction of NF-kB.

It has been suggested that TLR2 on DCs plays an important role in immune tolerance [26]. However, it was also showed that TLR2 on epithelial cells activates inflammatory mediators [6]. Thus, activation of TLR2, on the one hand, increases the immune tolerance favouring persistence of the bacterium in the stomach and, on the other hand, increases epithelial inflammatory responses resulting potentially in more severe gastritis [26]. However, Sun et al. observed enhanced gastric immunopathology in *H. pylori*-infected TLR2-KO mice, indicating that the impact of total TLR2 deficiency is greater on immune cells than on epithelial cells [26]. Therefore, it is tempting to speculate that the sialylation of *H. bizzozeronii* LPS may increase inflammatory responses by depressing the TLR2 response. However, further in vivo studies are needed for elucidating the proinflammatory effect of *Helicobacter* sialyl-LPS.

In conclusion, our study showed that the sialylation of *H. bizzozeronii* LPS in wild-type strains may modulate host immune response. Since we observed that the expression of sialylated LPS by *H. bizzozeronii* undergoes phase and phenotypic variation [11], changes in the stomach microenvironments due to diet, host-jump, antimicrobial treatment [12], may select subpopulations of *H. bizzozeronii* expressing or not sialylated LPS. These events could lead to an imbalance in the relationship between *H. bizzozeronii* and his host, underpinning the development of gastritis in both dogs and humans.

## Additional files

Additional file 1:
**LPS profile of wild type and mutant** ***H. bizzozeronii*** **CIII-1** ^**GEN**^ **and Heydar strains.** LPS profile in 15% TRIS-Glycine SDS-PAGE gel. *H. bizzozzeronii* strains CIII-1^GEN^ wild type and Heydar wild type showed low-molecular-weight LPS with a clear switch of the band after neuraminidase treatment (*) but no switch was observed in the relative *Δhbs2* isogenic mutants. Additional file 2:
**MALDI-TOF spectra of** ***H. bizzozeronii*** **CIII-1** ^**GEN**^ **wild type and Heydar wild type LPS.** (A) *H. bizzozeronii* CIII-1^GEN^. (B) *H. bizzozeronii* Heydar.

## Abbreviations

TLR: Tool-like Receptor
LPS: Lipopolysaccharide
PB: polymyxin-B
