# Supplementary figures and images for: Sialylation of Helicobacter bizzozeronii lipopolysaccharides modulates Toll-like receptor (TLR) 2 mediated response
Source: Vet Res. 2015 Jan 21;46(1):4. doi: 10.1186/s13567-014-0133-4 (PMC4299687; doi:10.1186/s13567-014-0133-4)

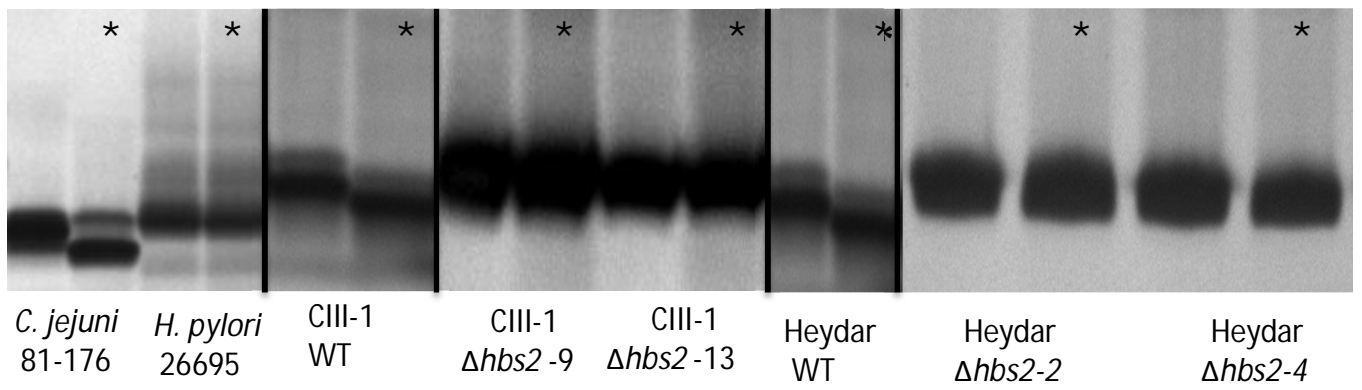

Supplement: Additional file 1: — LPS profile of wild type and mutant H. bizzozeronii CIII-1 GEN and Heydar strains. LPS profile in 15% TRIS-Glycine SDS-PAGE gel. H. bizzozzeronii strains CIII-1GEN wild type and Heydar wild type showed low-molecular-weight LPS with a clear switch of the band after neuraminidase treatment (*) but no switch was observed in the relative Δhbs2 isogenic mutants. [file 13567_2014_133_MOESM1_ESM.pdf]

A

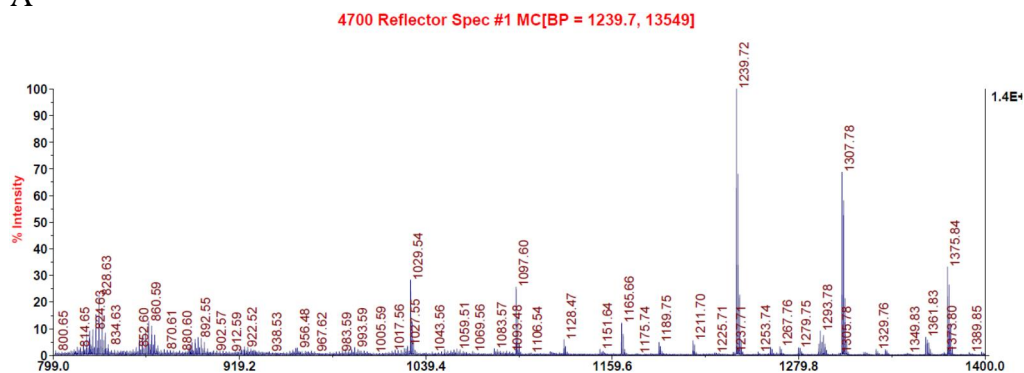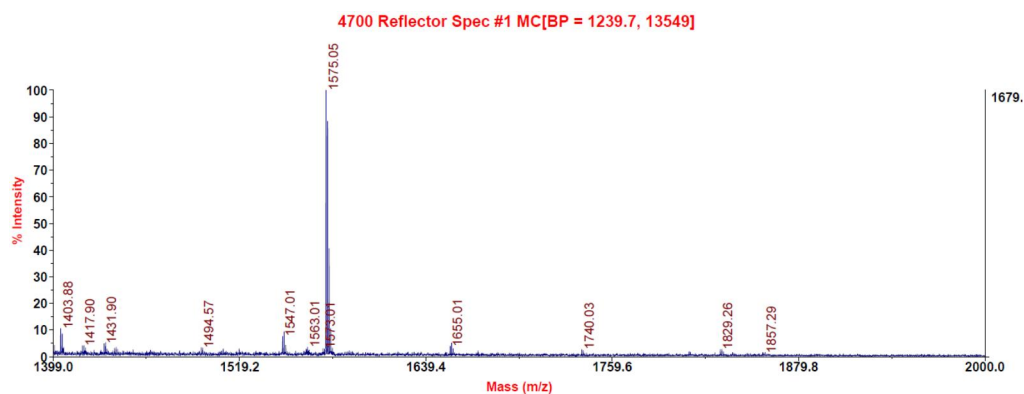

B

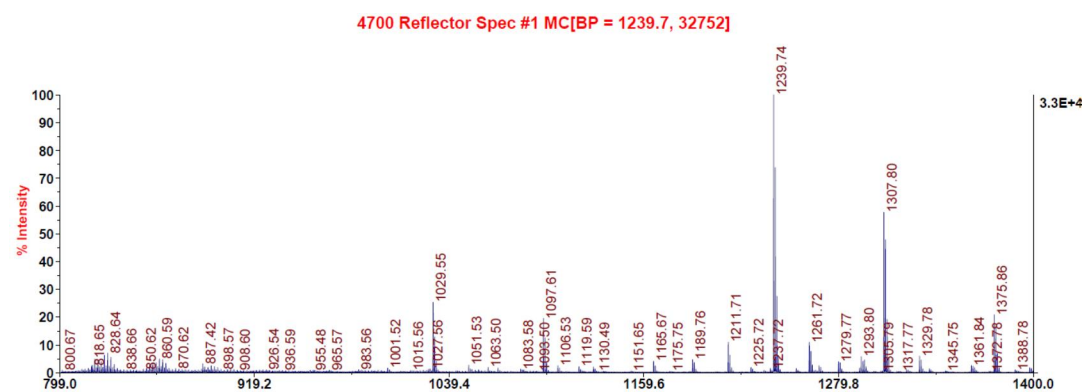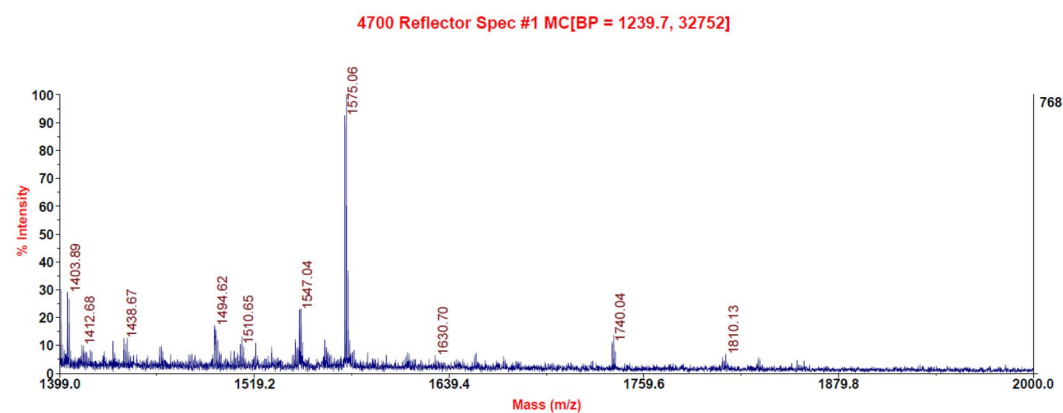

Supplement: Additional file 2: — MALDI-TOF spectra of H. bizzozeronii CIII-1 GEN wild type and Heydar wild type LPS. (A) H. bizzozeronii CIII-1GEN. (B) H. bizzozeronii Heydar. [file 13567_2014_133_MOESM2_ESM.pdf]
